# General Practices’ Experiences With Patients’ Web-Based Access to Medical Records: Survey Study

**DOI:** 10.2196/41832

**Published:** 2023-04-07

**Authors:** Jelle Keuper, Ronald Batenburg, Lilian van Tuyl, Robert Verheij

**Affiliations:** 1 Netherlands Institute for Health Services Research Utrecht Netherlands; 2 Tranzo Tilburg School of Social and Behavioral Sciences Tilburg University Tilburg Netherlands; 3 Department of Sociology Radboud University Nijmegen Nijmegen Netherlands

**Keywords:** patient access to records, electronic health record, patient portals, general practice, administrative burden, health information, shared decision-making, health care professionals

## Abstract

**Background:**

Patients’ web-based access to their medical records is expected to promote their role and responsibility in managing their own health and treatments and supporting shared decision-making. As of July 2020, general practices in the Netherlands are legally obliged to provide their patients access to their electronic medical records. Web-based access provision is facilitated and stimulated through a national support program named OPEN.

**Objective:**

We aimed to investigate general practice staff experiences with providing web-based access; investigate its impact on patient consultations, administrative actions, and patient inquiries; and investigate how it affects routine general practice workflow processes.

**Methods:**

In October 2021, a total of 3813 general practices in the Netherlands were invited to complete a web-based survey that included questions regarding their experiences with the provision of web-based access to medical records and how it affects routine general practice workflow. Responses of general practices that started providing web-based access before 2020, in 2020, or in 2021 were analyzed to identify trends.

**Results:**

Of 3813 invited general practices, 523 (13.72%) completed the survey. Approximately all responding general practices (487/523, 93.1%) indicated that they provide web-based access. Experiences with patients’ web-based access were diverse, with 36.9% (178/482) primarily positive, 8.1% (39/482) primarily negative, 42.3% (204/482) neutral, and 12.7% (61/482) could not (yet) indicate how they experienced web-based access. Of the total, two-thirds (311/473, 65.8%) reported an increase in e-consultations and a similar percentage (302/474, 63.7%) indicated an increase in administrative actions associated with web-based access provision. A small proportion of the practices (≤10%) experienced a decrease in patient contacts. Earlier adoption of web-based access was associated with a more positive attitude toward web-based access and more positive experienced effects related to patient contacts and general practice workflow.

**Conclusions:**

The surveyed general practices mainly experienced providing web-based access as either neutral or mostly positive, despite an increased number of patient contacts and administrative burden that were associated with its adoption. Periodic monitoring of experiences is needed to understand the temporal or structural nature of both the intended and unintended effects of patients’ web-based access to medical records for general practices and their staff.

## Introduction

### Background

Globally, there is a tendency in health care to stimulate patient empowerment, self-management, and shared decision-making (SDM) by providing individuals with access to their health data [1,2]. This tendency is strengthened by the increased use of health care technology and remote health care, which was especially visible during the COVID-19 pandemic [3]. Furthermore, providing patients access to their medical data is regarded as a solution to keep health care sustainable and improve health care processes. It is also believed that it enhances patient-physician communication. Patient access to health records is increasingly provided on the web, representing a relatively new opportunity to achieving these health care–related goals. Patient self-management is regarded as an important prerequisite to effectively use interventions such as patient web-based access [4]. Patient web-based access to health records has recently been introduced and investigated in several countries, including the United Kingdom, Australia, New Zealand, France, Norway, Sweden, Denmark, Estonia, and the United States, mainly stimulated through local or national governmental policies. The uptake and use of this service differ among countries [1,2,5-18]. Patient web-based access fits in a growing international movement advocating greater transparency in health care, including patient access to their health information and clinical notes, which originated in the United States (Boston) and is called OpenNotes. This movement aims to identify and disseminate best practices for sharing medical information with patients through research and education [19]. Thus far, perceived benefits of web-based access provision are improved patient satisfaction, patient empowerment, the facilitation of patient self-care participation, more patient self-control, improved communication, enhanced patient safety and security, increased medication adherence, and the facilitation of preventive care services. At the same time, patients expressed concerns regarding privacy issues, the lack of internet access, and user-friendliness [5,7,8,10,11,15,17,19-24]. From a clinician’s perspective, providing web-based access leads to stronger patient-physician relationships, enhanced trust, improved efficiency, and improved SDM. However, there are downsides to this perspective, such as concerns regarding the patient-physician relationship (eg, mistrust), secure access, safeguarding, user-friendliness, equitable access, costs, additional workload, and workflow issues [5,9,11,14,23-25]. With regard to the impact of web-based access provision on the organization’s workload, several reviews report an inconsistent effect on the number of telephone consultations, email consultations, and face-to-face consultations [5,11,18,24]. Some studies included in these reviews found a decrease, whereas others found an increase or no change in the number of consultations.

Since July 2020, general practices in the Netherlands have been legally required to provide patients aged ≥16 years electronic access to their medical records if requested. Electronic access means that general practices can provide either (1) a digital copy of the patient’s medical record or (2) web-based access to the patient’s medical record; for example, through the use of a patient portal or a so-called personal health environment. In the Netherlands, a personal health environment is an application that individuals can voluntarily use to collect, access, manage, and share their personal health data from various health care providers in a private and secure digital environment. This term is similar to the term personal health record, which is mainly used in other publications. In the Netherlands, web-based access to the general practice’s medical record includes patient access to their complete medical records, including information about diagnoses, medication, allergies, laboratory test results, and evaluation and treatment plan notes. However, notes made by the health care professional and data that can harm the privacy of other persons are excluded. This Dutch legal regulation is intended to make patients’ health data available to make it easier for patients to view and manage their medical data securely. For health care professionals, the aim is to help them easily access the correct and most up-to-date medical information to support the patient’s treatment and minimize medical errors [26].

In 2019, the Dutch general practice associations initiated a support program named OPEN to help general practices provide web-based access through financial, organizational, and educational support, for which governmental funding was provided. Similar to the international OpenNotes movement, the Dutch OPEN initiative stimulates scientific research about patients’ web-based access to their health records to create and expand the knowledge about the impact of this service on general practice care. Among the activities initiated by the OPEN program is scientific research by a consortium involving Radboud University, Nijmegen, Netherlands; Maastricht University, Maastricht, Netherlands; and the Netherlands Institute for Health Services Research (Nivel), Utrecht, Netherlands. The OPEN program was implemented through regional coalitions, in which at least 2 regional general practice organizations participate [27]. Through these regional coalitions, the program aims to align the provision of web-based access with the regional needs of general practices. It is expected that web-based access will facilitate patients to (1) be better informed regarding their own health, (2) be better prepared for a practice consultation, (3) have a more efficient patient-physician conversation, and (4) be more involved in their own care. It is expected that this will promote patient self-control and SDM, which will ultimately improve the quality of health care for patients.

### Objectives

The main objective of our study was to investigate general practice staff experiences with web-based access. Specifically, the following questions were addressed:

1. How many general practices provide patient web-based access and since when?
2. What are general practice staff experiences with providing web-based access?
3. What is the impact of web-based access provision on patient consultations, administrative actions, and patient questions?
4. How does web-based access affect general practice workflow routines?

Providing insight into the answers to these research questions will generate valuable information for the further successful development of web-based access to patients’ medical records.

We hypothesized that general practices with longer experience in providing web-based access will perceive this as more positive and less burdening than practices with less experience in providing web-based access. One of the underlying arguments for this expectation is that these practices voluntarily adopted web-based access before July 2020, when web-based access became obligatory. This group of early adopters was most likely intrinsically motivated to adopt this service and had more time to perceive the positive effects of this intervention. Furthermore, adopting web-based access in one’s practice requires time to install and get used to it before its proposed benefits emerge, according to the diffusion of innovation theory by Rogers et al [28]. In our analyses, we consequently distinguished practices that adopted web-based access before 2020, in 2020, and in 2021.

To the best of our knowledge, our study will provide first insights into the perceived intended and unintended effects of web-based access in the first years after its introduction. It will also provide general practice implications and key points of attention for further successful development of this intervention.

## Methods

### Setting: Study Participants

In October 2021, a web-based questionnaire was sent to 3813 general practices in the Netherlands. Contact details of general practices were obtained from Nivel’s Healthcare Professionals Registries [29]. One staff member in each general practice was asked to complete the survey on behalf of the practice; in most cases, this was the practice manager or practice owner [3]. In the Netherlands, general practice owners have completed medical education and are practicing general practitioners (GPs). The practice manager is mainly responsible for personnel management tasks and is usually not trained medically. Furthermore, general practices in the Netherlands vary in terms of skill mix and personnel.

### Study Design: Web Survey

The web-based survey was developed by the research team and contained closed-ended questions: (1) if general practices already provide web-based access; (2) if so, the year of web-based access adoption; (3) the experiences of general practice staff with its provision; (4) the effects of web-based access on the number of patient contacts and administrative actions; (5) its impact on routine general practice workflow; (6) its impact on general practice staff workload; (7) the associated patient instruction time; and (8) the type of patient questions about web-based access use. The respondents were asked to estimate these items in general practice. They were able to explain their answers through 2 open-ended questions. In addition, they could indicate other changes they perceived because of web-based access by completing the answer option *other (changes), namely*. Before the survey was distributed in October 2021, it was reviewed and pretested by 4 other researchers, 2 GPs, and 1 policy maker. For each general practice, a personalized web link to the web survey was generated through Nivel’s Healthcare Professionals Registries [29]. Each general practice received an email containing this personalized web link to the questionnaire, which was completed by 1 respondent per practice. To increase attention to this study and the surveys, 2 reminder emails and 2 reminder messages using the social media accounts of the research institute were sent in October and November 2021. These web survey questions can be found in Multimedia Appendix 1.

### Data Collection

The survey data were collected anonymously and provided to the researchers for further analysis. Consequently, the names of the general practices and names of practice staff were not available for this study’s researchers.

### Ethical Considerations

Per Dutch legislation (Medical Research Involving Human Subjects Act), ethics approval by a medical ethics committee was not required for this study because the research participants were not subjected to interventions and no rules of behavior were imposed on them [30]. Consequently, we did not include an institutional or external review board statement for this study.

### Data Analysis

Data analysis was performed using Stata (version 16; StataCorp LLC) and Excel (version 2019; Microsoft Office). Descriptive statistics were used to report (1) how many general practices provided web-based access, (2) their experiences with the provision of web-based access, and (3) their effects on general practice workflow and staff workload. For in-depth analysis and to test our hypothesis, the response group was categorized into 3 subgroups based on the year a general practice started providing web-based access: before 2020 (group 1), in 2020 (group 2), and in 2021 (group 3). A chi-square test of independence was performed to test differences in the responses between these 3 subgroups. A *P* value ≤.05 was regarded as significant.

## Results

### Use of Patient Web-Based Access

In total, 13.72% (523/3813) of invited general practices participated in this study. The composition of the response group was similar to the composition of the invited group of general practices with regard to the type of general practice (solo, duo, or group practice) and region. Approximately all responding practices (487/523, 93.1%) provided their patients with web-based access to their medical records, mainly through the OPEN program. This percentage was similar to the percentage of all the general practices that participated in the OPEN program at that moment, which is monitored continuously through the OPEN program. A small proportion of practices (36/523, 6.9%) indicated that they did not provide web-based access yet. Regarding the year of web-based access adoption, 52.7% (238/452) indicated that they started with web-based access provision in 2020, 38.1% (172/452) in 2021, and 9.3% (42/452) before 2020 (Table 1).

**Table 1:** Provision of web-based access by general practices and year of web-based access adoption.

Provision of web-based access by general practices			Values, n (%)
**Do you currently provide patients web-based access to their medical record? (n=523)**			
	Yes, through the OPEN program	469 (89.7)	
	Yes, on own initiative	18 (3.4)	
	No, not yet, but I am planning to provide patients web-based access	36 (6.9)	
	Total	523 (100)	
**If a practice provides web-based access, it started^a^ (n=452):**			
	Before 2020	42 (9.3)	
	In 2020	238 (52.7)	
	In 2021	172 (38.1)	
	Total	452 (100)	

^a^The number in the total row is not equal to the sum of the respondents who answered *Yes* to the first question, because not all respondents (35 missing) indicated when they started providing web-based access.

### Experiences of General Practice Staff

On the basis of a 4-point scale, one-third of the practice respondents (178/482, 36.9%) was mostly positive about web-based access provision, whereas slightly more respondents (204/482, 42.3%) answered *neutral* to this question, and 8.1% (39/482) were mostly negative. The remaining 12.7% (61/482) indicated that they did not know (yet) what their experiences with web-based access were (Table 2).

In line with our hypothesis, responding practices that had more experience with providing web-based access (ie, adoption before 2020) were more often positive about this (23/41, 56%) than practices that adopted it in 2020 (97/236, 41.1%) and after 2020 (46/169, 27.2%). The percentages significantly differed among these 3 subgroups (*P*<.001). When leaving out the answer option *I do not know (yet)*, this was not significantly different (*P*=.17; Table S1 in Multimedia Appendix 2).

Respondents were also asked how their colleagues, who also have experience with the provision of web-based access, perceived this (ie, GPs, general practice assistants, practice nurses in somatic health care, practice nurses in mental health care, and practice managers). The results show similar findings compared with the respondent’s own experience. This means that, according to the respondent, colleagues also perceive the provision of web-based access as neutral or primarily positive. However, a large share of the respondents (18.5%-58%) answered that they did not know (yet) how their colleagues perceived this (Table 2).

For all the specified functions, respondents from practices that already started with providing web-based access before 2020 (group 1) also more often indicated that their colleagues were mostly positive about it. In contrast, surveyed practices that started providing web-based access in 2021 (group 3) more often indicated *I do not know (yet)* compared with surveyed practices that already provided this before 2020 (group 1) or in 2020 (group 2). When leaving out the answer option *I do not know (yet)*, we found slightly different results for a few general practice staff functions (Table S1 in Multimedia Appendix 2).

Furthermore, 70.1% (338/482) of respondents explained their answers through an open-ended question provided in the survey. These answers mainly concerned that general practice staff experiences are associated with (1) aspects of the use of web-based access, (2) the impact of web-based access provision on general practice staff workload, (3) organizational and technological aspects of web-based access provision, and (4) the changed patient-physician relationship.

First, with regard to the use of web-based access, some respondents answered that they promoted its use among their patients and are happy with it. In contrast, others indicated that almost none of their patients use it or that only individuals with a higher level of education (without illnesses) are primarily using it. In addition, it might not be suitable for patients with poor digital skills. Second, the respondents indicated varying results regarding the impact of web-based access on workload in general practice. Some respondents reported an increase because of patient inquiries and IT-related questions, whereas a smaller share indicated that it did not affect or improve their workload. Third, according to the respondents, general practice staff experiences depend on the proper functioning of web-based access (eg, if it fits well into routine general practice workflow processes) and sufficient (financial) support. Finally, respondents indicated that providing web-based access has changed their role as health care professionals, stimulating patients’ self-management and shared responsibility for a patient’s well-being, which can sometimes be difficult for physicians. Some respondents also indicated that they are occasionally worried if their patients can correctly interpret the information in their medical records.

**Table 2:** Staff experiences with the provision of web-based access (categorized by year of adoption).

Staff experiences		Overall sample^a^, n (%)	Group 1 (<2020), n (%)	Group 2 (2020), n (%)	Group 3 (2021), n (%)	Test results					
						Chi-square (*df*)		*P* value		N	
**Respondent^b^ (overall sample: n=482; group 1: n=41; group 2: n=236; group 3: n=169)**							41.0 (6)		<.001		446
	Mostly positive	178 (36.9)	23 (56.1)	97 (41.1)	46 (27.2)						
	Mostly negative	39 (8.1)	3 (7.3)	19 (8.1)	14 (8.3)						
	Neutral	204 (42.3)	14 (34.1)	107 (45.3)	69 (40.8)						
	I do not know (yet)	61 (12.7)	1 (2.4)	13 (5.5)	40 (23.7)						
	Total	482 (100)	41 (100)	236 (100)	169 (100)						
**General practitioner (overall sample: n=475; group 1: n=41; group 2: n=232; group 3: n=168)**							32.3 (6)		<.001		441
	Mostly positive	174 (36.6)	23 (56.1)	92 (39.7)	46 (27.4)						
	Mostly negative	33 (6.9)	3 (7.3)	16 (6.9)	10 (6)						
	Neutral	180 (37.9)	12 (29.3)	97 (41.8)	61 (36.3)						
	I do not know (yet)	88 (18.5)	3 (7.3)	27 (11.6)	51 (30.4)						
	Total	475 (100)	41 (100)	232 (100)	168 (100)						
**General practice assistant (overall sample: n=480; group 1: n=40; group 2: n=236; group 3: n=169)**							34.9 (6)		<.001		445
	Mostly positive	148 (30.8)	22 (55)	80 (33.9)	37 (21.9)						
	Mostly negative	46 (9.6)	7 (17.5)	23 (9.7)	14 (8.3)						
	Neutral	155 (32.3)	8 (20)	83 (35.2)	53 (31.4)						
	I do not know (yet)	131 (27.3)	3 (7.5)	50 (21.2)	65 (38.4)						
	Total	480 (100)	40 (100)	236 (100)	169 (100)						
**Practice nurse in somatic health care (overall sample: n=478; group 1: n=40; group 2: n=236; group 3: n=168)**							22.2 (6)		<.001		444
	Mostly positive	142 (29.7)	20 (50)	72 (30.5)	40 (23.8)						
	Mostly negative	15 (3.1)	3 (7.5)	8 (3.4)	3 (1.8)						
	Neutral	143 (29.9)	8 (20)	80 (33.9)	46 (27.4)						
	I do not know (yet)	178 (37.2)	9 (22.5)	76 (32.2)	79 (47)						
	Total	478 (100)	40 (100)	236 (100)	168 (100)						
**Practice nurse in mental health care (overall sample: n=471; group 1: n=41; group 2: n=235; group 3: n=163)**							16.1 (6)		.01		439
	Mostly positive	73 (15.5)	14 (34.1)	38 (16.2)	17 (10.4)						
	Mostly negative	13 (2.7)	2 (4.9)	6 (2.6)	5 (3.1)						
	Neutral	112 (23.8)	6 (14.6)	60 (25.5)	38 (23.3)						
	I do not know (yet)	273 (58)	19 (46.3)	131 (55.7)	103 (63.2)						
	Total	471 (100)	41 (100)	235 (100)	163 (100)						
**Practice manager (overall sample: n=450; group 1: n=41; group 2: n=222; group 3: n=156)**							13.1 (6)		.04		419
	Mostly positive	101 (22.4)	15 (36.6)	53 (23.9)	27 (17.3)						
	Mostly negative	18 (4)	4 (9.8)	8 (3.6)	6 (3.8)						
	Neutral	107 (23.8)	5 (12.2)	56 (25.2)	37 (23.7)						
	I do not know (yet)	224 (49.8)	17 (41.4)	105 (47.3)	86 (55.1)						
	Total	450 (100)	41 (100)	222 (100)	156 (100)						

^a^The percentages and numbers in the overall sample column are not equal to the sum of the other 3 columns (based on the year of adoption) because not all respondents indicated when they started providing web-based access.

^b^From previous studies, we know that the respondent is generally a practice manager or practice owner.

### Effects on General Practice Workflow Processes

**Figure 1:**
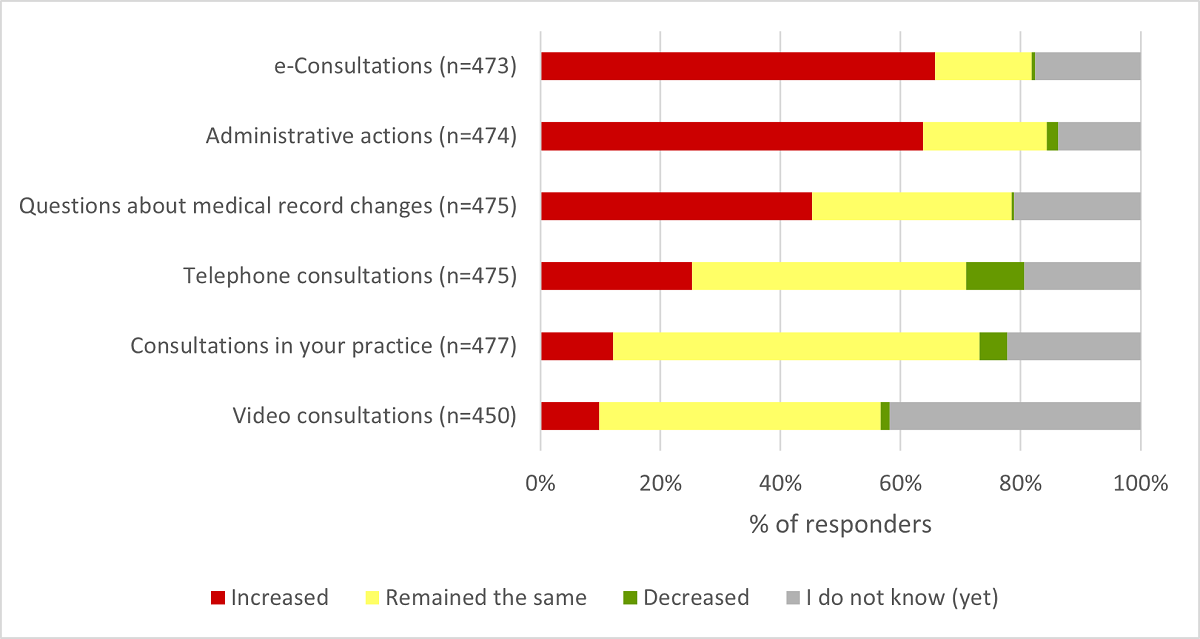
Effects of web-based access on the number of consultations, administrative actions, and patient questions in general practices.

One of the intended effects of web-based access is the improvement of general practice workflow. Our survey results showed that 65.8% (311/473) of the respondents reported an increase in the number of e-consultations because of web-based access. A similar percentage (302/474, 63.7%) indicated an increased administrative burden owing to web-based access, which contradicts the expected workflow improvement. Furthermore, 45.3% (215/475) of the practices responded that patient questions about medical record amendments increased (Figure 1). Only a few practices indicated that these numbers decreased because of web-based access provision.

Regarding the year of web-based access adoption, these results differ significantly among the 3 subgroups of practices, as well as for the other survey items shown in Figure 1 (e-consultations, *P*<.001; administrative actions, *P*=.004; patient questions about medical record changes, *P*<.001; telephone consultations, *P*<.001; consultations in your practice, *P*<.001; video consultations, *P*<.001; Table S2 in Multimedia Appendix 2). Especially for the number of e-consultations (36/40, 90%) and administrative actions (31/40, 78%), group 1 practices more often indicated that these numbers increased owing to the provision of web-based access than group 2 and group 3 practices. However, these group 1 practices more often answered that the number of consultations in their practice (3/40, 8%) and the number of telephone consultations (5/39, 13%) decreased because of web-based access provision. For group 2 practices, these percentages were 5.6% (13/233) and 9.5% (22/231), respectively, and for group 3 practices, these percentages were 3% (5/169) and 8.8% (15/170), respectively.

**Figure 2:**
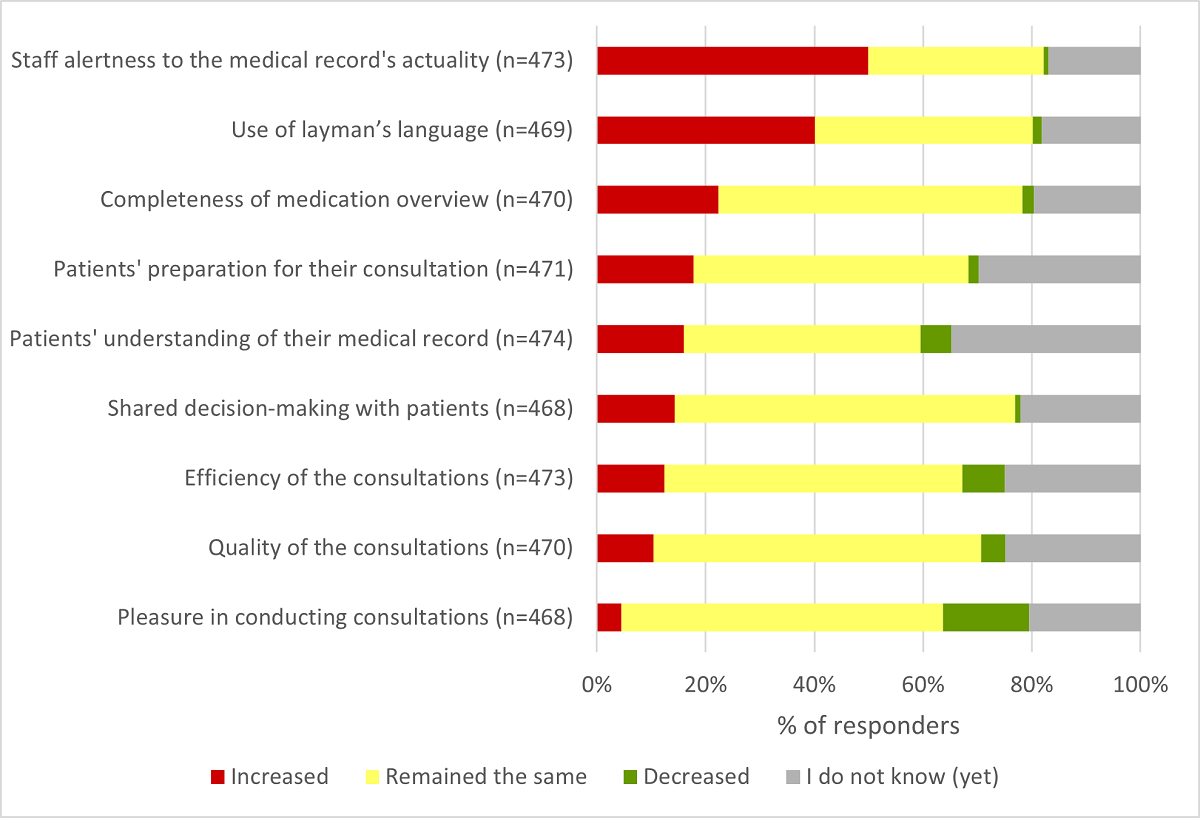
Effects of web-based access on specified general practice workflow processes.

Another intended effect of web-based access is improving patient-professional communication, as it can be a base for trust, transparency, and SDM. In our survey, half of the practice respondents (236/473, 49.9%) specified that their alertness to the accuracy of the medical record data increased because of the provision of web-based access. In addition, almost 40.1% (188/469) of the respondents started using *layman’s language* more often (Figure 2).

The most notable results of the in-depth analyses are that early adapters of web-based access (1) more often answered that the extent to which SDM is applied to their patients increased (10/40, 25%) and (2) more often indicated that the efficiency of the consultations decreased (6/40, 15%) than the participating practices that adopted it in 2020 or 2021. Except for the answer category *other changes* (*P*=.72), for all other survey items shown in Figure 2, answers differed significantly among the 3 subgroups of practices (staff alertness, *P*=.02; layman’s language, *P*=.001; completeness of medication overview, *P*=.001; patients’ preparation for their consultation, *P*<.001; patients’ understanding of their medical record, *P*<.001; shared decision-making, *P*=.001; efficiency of consultations, *P*<.001; quality of consultations, *P*<.001; and pleasure in conducting consultations, *P*=.001; Table S3 in Multimedia Appendix 2). Other changes that the respondents specified were mainly related to (1) medical records management, (2) patients being more demanding, (3) providing patient with instructions regarding web-based access, (4) disadvantaged patients not using web-based access, and (5) IT-related questions.

### Effects on General Practice Staff Workload

**Figure 3:**
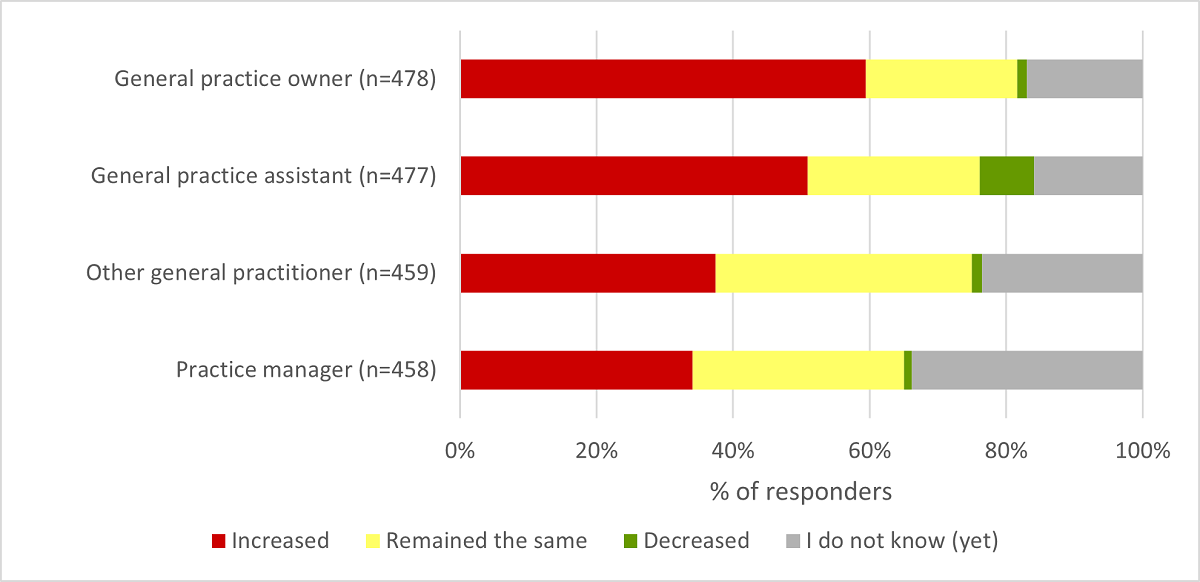
Effects of web-based access on time burden in general practices, specified by function.

The additional administrative workload appears to be associated with the provision of web-based access, particularly for responding practices that recently started with this. In addition, our survey shows that providing web-based access is associated with an increased time burden for general practice assistants and practice owners, according to a small majority of the respondents (243/477, 50.9% and 284/478, 59.4% of the respondents, respectively; Figure 3).

The in-depth analysis also showed that practices that already adopted web-based access before 2020 (group 1) more often indicated that the time burden has increased for these 2 functions compared with practices that started more recently (group 2 and group 3; *P*<.001; Table S4 in Multimedia Appendix 2). This was also the case for the functions *other GPs* and the *practice manager* within general practices (*P*=.002 and *P*=.03, respectively). However, group 1 practices also more often indicated that the time burden for general practice assistants has decreased. For these 4 questions and the questions shown in Figures 1 and 2, it should be noted that practices that started more recently (group 3) were relatively more likely to fill in *I do not know (yet)*.

### Patient Instruction Time

Respondents indicated that they spent an average of 6.7 (SD 5.43) minutes per patient explaining how to use web-based access when a patient wanted to use it for the first time. About one-fifth of the respondents (104/471, 22.1%) estimated that they spent ≥10 minutes instructing patients on how to use it. A relatively large group of respondents (177/471, 37.6%) could not (yet) provide an estimate of the instruction time involved (Table 3).

**Table 3:** Estimated patient instruction time (categorized) for the use of web-based access.

Patient instruction time		Values, n (%)	Values, mean (SD)^a^
**Duration of instruction per patient (minutes)**		471 (100)	6.7 (5.43)
	0-4	98 (20.8)	1.7 (1.1)
	5-9	92 (19.5)	5.1 (0.6)
	10-14	66 (14)	10 (0.0)
	15-19	25 (5.3)	15 (0.0)
	≥20	13 (2.8)	21.7 (3.9)
	I do not know (yet), I cannot (yet) estimate this	177 (37.6)	—^a^

^a^Mean (SD) was based on the responses of 294 respondents. Respondents who answered *I do not know (yet), I cannot (yet) estimate this* were not included in calculating the mean and SD.

### Patient Contacts About Web-Based Access

Patients contacting their practice about web-based access to their medical records, mainly had questions about diagnostic test results (336/482, 69.7%), episode lists (230/482, 47.7%), the medication overview (206/482, 42.7%), and how web-based access works (201/482, 41.7%), according to the respondents (Table S5 in Multimedia Appendix 2). An episode list is an overview of a patient’s health problems at one point in time. Approximately 6.8% (33/482) of the respondents answered *other, namely* here and indicated that they received questions about various other matters.

First, these concern practical questions about obtaining web-based access, having difficulties logging in, being unable to find something in their medical records, and receiving notifications from their patient portal. Second, there were questions about the health record content: presumed incompleteness or completeness, contraindications, photos of skin conditions, and medical matters. Third, there were questions regarding the use of health records for legal purposes, making appointments, deleting things that have been noted, and confusion regarding the type of consultation.

Finally, for this question, there were some differences among group 1, group 2, and group 3 practices: practices with a longer history of web-based access provision indicated relatively more often that their patients contacted them about one of the specified categories shown in Table S5 in Multimedia Appendix 2, except for the categories *I do not know (yet), I cannot say (yet)* and *other, namely*. Group 3 practices more often indicated these categories (Table S5 in Multimedia Appendix 2).

Furthermore, 29.3% (141/482) of respondents explained their answers through an open-ended question provided in the survey. These answers mainly concerned that (1) the number of patient questions about web-based access increased; (2) the service did not (always) work well, leading to staff dissatisfaction; (3) web-based access has led to an increased workload; (4) web-based access is almost not used by their patients and that patients consequently did not (yet) provide feedback about it; (5) web-based access is not suitable for all patients and that patients sometimes have difficulties using web-based access; and (6) patients mainly contact the general practice about medical record management, such as changing their health data or medication overview.

## Discussion

### Relevance and Principal Findings

This study provides insight into the experiences of 13.72% (523/3813) of the general practices in the Netherlands with patient web-based access to their medical records. Many of the surveyed general practice staff experienced patient web-based access as neither positive nor negative. Among the remaining respondents, the largest share was mostly positive. Only a small share perceived it as mostly negative. These results align with those of related studies, which found that general physicians are more often positive than negative regarding providing web-based access [31-33]. However, some variations in the results have been reported among medical specialties and countries [33].

In line with our hypothesis, early adopters (group 1 practices) reported more positive experiences with web-based access provision than late adopters (group 2 or group 3 practices). These findings are consistent with results from Sweden, where Scandurra et al [34,35] found that health care professionals already familiar with web-based access provision are more positive regarding this than health care professionals who have not yet introduced this service. Another study by Keplinger et al [36] found that physician work satisfaction increased during the implementation of a web-based patient portal compared with the period immediately before implementation. This indicates that physician attitudes changed positively over time. It was not possible in our study, or in any of the included literature, to distinguish between a possible selection effect or causation effect. Causation would mean that the early adopter’s positive attitude results from their long experience with web-based access provision. A selection effect would indicate that early adopters were already more positive about digital innovations, including patient web-based access.

In our study, a relatively large proportion of respondents indicated that they did not experience any changes because of web-based access provision. Furthermore, a relatively large group replied that they did not know (yet) the effects of web-based access on their practice and practice staff. The latter answer was particularly given by practices that recently started with web-based access provision and could not yet estimate how web-based access provision influenced the practice’s workflow processes. Surveyed practices that adopted web-based access before 2020 were better able to assess its effects. Following the theory of the diffusion of innovations by Roger et al [28], it might be conceivable that group 2 and group 3 practices will also experience similar positive effects after a couple of years when web-based access is also better integrated into the general practice’s workflow. However, we should also note that we did not find substantial differences in respondents’ experiences among practices with longer or shorter implementation history when omitting the answer option *I do not know (yet)*. This may imply that there are only a few differences among the 3 subgroups of practices regarding how they experienced the provision of web-based access. However, we chose to include this answer option, which was indicated by a relatively large number of respondents and cannot be neglected in our results.

Furthermore, a substantial proportion of the surveyed general practices experienced several unintended effects of web-based access provision, including additional workload, which various respondents also mentioned in the open-ended questions. In our study, more than half of the practice representatives indicated extra time burden for the GP owner or owners and practice assistant or assistants. In addition, many practices mentioned an increase in the number of administrative actions. Furthermore, a small proportion experienced less pleasure in conducting consultations because of the provision of web-based access.

Other notable findings included that about two-thirds of the respondents experienced an increase in the number of e-consultations and that almost half of the respondents indicated an increase in the number of questions from patients about changes to their medical records. Similar research from other countries, where web-based access was also introduced recently, showed that this could lead to an increase in the practice’s workload and the number of patient questions related to web-based access to their medical record [37,38]. For example, a study by Palen et al [37] found that web-based access use was associated with an increase in clinical services. Another study by Miller et al [39] showed that patient portals, including web-based access, tend to create a high volume of patient messages and additional time pressure. Turner et al [40] also reported increased practices’ workload as a consequence of web-based access provision to patient medical records. By contrast, a study by Delbanco et al [41] showed that only a few physicians reported additional workload in their practice because of the provision of web-based access, with regard to the number of e-consultations, longer visits, more time to address patients’ questions outside of office hours, changing documentation content, and a need for more time to write notes. Contrary to our findings, a study by Fitton et al [42] showed that providing web-based access can lead to fewer appointments and telephone calls. As described earlier, literature reviews reported contradictory results regarding the impact of web-based access provision on an organization’s workload [5,11,18,24].

Furthermore, we found that a small share of practice representatives indicated that web-based access led to several positive intended effects, including a decrease in telephone consultations. This was more common among group 1 practices compared with the other practices. However, a larger share of all practices indicated that telephone consultations increased. This might be explained by the fact that web-based access is new to patients, leading to additional questions about its use, mainly via telephone contact with their general practice. In addition, we found that a large share of general practices is more alert to the up-to-dateness of the patient’s medical record and that they are using more layman’s language. Furthermore, a small share of the surveyed practices indicated that patients’ understanding of their medical records and SDM with patients increased, and the efficiency and quality of the consultations improved because of web-based access. In addition, almost one-quarter of the respondents indicated that the completeness of the medication overview increased, which was also an intended effect of web-based access provision and is positive for both the patient and health care professional.

From the general practice perspective, a notable positive effect of web-based access provision is that some respondents indicated that it led to decreased time burden for general practice assistants. This was especially the case for early adopters (group 1), who already have more experience with providing web-based access. If this pattern continues in the future, providing web-based access might also have a work pressure–reducing effect on general practice staff, especially for general practice assistants, who are particularly the first point of contact for patients. The study by Miller et al [39] tends to agree with many of these findings. In their interview study, participants reported (potential) benefits, including fewer phone calls, handling messages more quickly, increased patient ability to manage their health, and medical error reductions [39]. In contrast, research by Turner et al [40] recently showed that web-based access provision could also negatively impact patients’ understanding of their health care, the quality of their medical records, and patient safety.

When balancing the intended and unintended effects, we can conclude that providing web-based access has generally led to an increase in general practices’ workload. It has also improved the quality of the patients’ medical records and their understanding of medical records. Periodic monitoring of experiences is required to understand the temporal or structural nature of both the intended and unintended effects of patients’ web-based access to medical records.

### Strengths and Limitations

To the best of our knowledge, this study is the first to evaluate general practices’ experiences with providing web-based access in the Netherlands. We investigated these experiences at an early stage, as almost all Dutch general practices only recently provide patient web-based access. A strength of this study is that we collected large-scale data from a representative sample of >500 Dutch general practices based on the following practice characteristics: region, practice type (solo, duo, or group practice), and the adoption rate of web-based access. Another strength of this study is that we compared experiences of early adopting practices with general practices that only recently started providing web-based access to their patients. This makes it possible to make cautious predictions regarding the future effects of web-based access provision.

This study has several limitations. Each survey was completed by 1 practice representative who answered all questions on behalf of the practice, which could have led to response bias. Results might have been different when another colleague within the general practice filled out the survey. Ultimately, it might have been difficult for the respondent to correctly estimate the effects of web-based access provision for their colleagues. The respondents in our surveys were mainly practice owners and practice managers. In general, these 2 functions have the best position to estimate the staff experiences of their colleagues, as they are generally responsible for the overall practice personnel management. However, we had a representative sample of general practices (based on region, practice type, and adoption rate of web-based access), and the response rate (523/3813, 13.72%) was relatively low. We could not check whether a selection of general practices with interest in the topic had a higher likelihood of having an opinion on this subject. Estimating the impact of this potential selection bias and response bias on the study results is difficult. Furthermore, this study was conducted during the COVID-19 pandemic, when Dutch COVID-19 government measures were relatively strict. As a large share of general practices started providing web-based access during the pandemic, it might have been difficult for respondents to estimate the *net* impact of providing web-based access for their general practice.

### Implications for Future Research

The results of this study imply that, in addition to the intended effects of web-based access provision, it may also lead to additional workload in general practices. Consequently, with regard to the increasing shortages in health care workforce in Dutch general practice, it is vital to pay more attention to such unintended effects and investigate what causes these effects and how they can be reduced. When new health care innovations or technologies are being implemented, the aim should be to implement technology that does not increase general practices’ workload or can be easily integrated within the existing general practice workflow. Although we included a few open-ended questions, which provided more in-depth insights into the experiences of general practice staff with web-based access, performing qualitative research with relevant stakeholders (eg, general practice staff, related health care organizations, governmental institutions, and policy makers) will provide more insights into the barriers to and facilitators of successful web-based access provision in general practices in the Netherlands. In addition, this can contribute to a better understanding of which general practice staff is usually responsible for instructing patients and answering patients’ questions regarding web-based access to their medical records.

Furthermore, our study suggests that a small group of practices (36/523, 6.9%) still does not provide web-based access. Providing more insight into the backgrounds and obstacles experienced in this group of practices might be interesting for future research.

Finally, it is important to investigate how patients in the Netherlands perceived the mandatory provision of web-based access in general practice. This is also a research goal of the scientific research activities of the OPEN program [43], as this intervention is intended to stimulate patient empowerment, self-management, and SDM.

### Implications for General Practice

The intended and unintended effects of web-based access provision might have implications for general practices. We found that a large share of the responding general practices indicated increased time burden for general practice staff. Patient instruction time took, on average, approximately 7 minutes per patient. In the Netherlands, consultation time in general practices normally takes about 10 minutes for one health-related problem, which implies that instruction time is a major investment for general practices. Therefore, staff members could adopt more efficient ways to give their patients instructions about using web-based access, such as providing instructions to groups of patients at one time or by presenting information (videos and instructions) on the general practice’s website. As 41.7% (201/482) of the surveyed general practices indicated that patients contacted them about how web-based access works, such interventions might also lead to a decrease in patient contacts.

Because patient instruction takes a long time and many general practices answered that patients contacted them with questions about web-based access to their medical records, this might indicate that groups of patients have difficulties understanding their medical records or using digital applications. The intended effects of web-based access, including improved quality of patients’ medical records and a more equal patient-physician relationship, which were found in this and other studies, might not reach patients who are not digitally proficient or do not have sufficient health literacy to adequately understand their medical records. Consequently, the government, general practices, and other stakeholders could think about how web-based access to medical records can also benefit these patient groups and how this could be provided alternatively or more simply. The fact that a large share of general practices answered that they started using more *layman’s language* and that staff alertness to the medical record’s up-to-dateness has increased might indicate that general practices are already aware of this issue.

### Conclusions

Most of the surveyed general practices experienced providing web-based access as either neutral or mostly positive. Only a small share of practices experienced it as mostly negative. It generally improved the quality of the medical records and patients’ understanding of their medical records and promoted an equal patient-physician relationship. It has also led to an increase in the number of workflow processes and general practices’ workload. As a large share of general practices only recently adopted web-based access and could not (yet) indicate how they perceived its impact, the effects of its adoption might change in the coming years. Future research and policy should focus on how this intervention develops further, how the intended effects can be maximized, and how the unintended negative effects can be minimized.

## Appendix

Multimedia Appendix 1 Web-based survey questions.

Multimedia Appendix 2 In-depth analyses regarding staff experiences with web-based access and effects of web-based access on general practice.

## Abbreviations

GP: general practitioner
Nivel: Netherlands Institute for Health Services Research
SDM: shared decision-making

## References

[1] Nøhr C, Parv L, Kink P, Cummings E, Almond H, Nørgaard JR, Turner P (2017). Nationwide citizen access to their health data: analysing and comparing experiences in Denmark, Estonia and Australia. BMC Health Serv Res.

[2] Benjamins J, Haveman-Nies A, Gunnink M, Goudkuil A, de Vet E (2021). How the use of a patient-accessible health record contributes to patient-centered care: scoping review. J Med Internet Res.

[3] Keuper J, Batenburg R, Verheij R, van Tuyl L (2021). Use of e-health in Dutch general practice during the COVID-19 pandemic. Int J Environ Res Public Health.

[4] Bodenheimer T, Wagner EH, Grumbach K (2002). Improving primary care for patients with chronic illness: the chronic care model, Part 2. JAMA.

[5] Mold F, de Lusignan S, Sheikh A, Majeed A, Wyatt JC, Quinn T, Cavill M, Franco C, Chauhan U, Blakey H, Kataria N, Arvanitis TN, Ellis B (2015). Patients' online access to their electronic health records and linked online services: a systematic review in primary care. Br J Gen Pract.

[6] Dendere R, Slade C, Burton-Jones A, Sullivan C, Staib A, Janda M (2019). Patient portals facilitating engagement with inpatient electronic medical records: a systematic review. J Med Internet Res.

[7] de Lusignan S, Ross P, Shifrin M, Hercigonja-Szekeres M, Seroussi B (2013). A comparison of approaches to providing patients access to summary care records across old and new Europe: an exploration of facilitators and barriers to implementation. Stud Health Technol Inform.

[8] Rahbek Nørgaard J (2013). E-record - access to all Danish public health records. Stud Health Technol Inform.

[9] Janssen A, Keep M, Selvadurai H, Kench A, Hunt S, Simonds S, Marshall T, Hatton L, Dalla-Pozza L, McCullagh C, Shaw T (2021). Factors that influence use of a patient portal by health professionals. Int J Environ Res Public Health.

[10] Hannan A (2010). Providing patients online access to their primary care computerised medical records: a case study of sharing and caring. Inform Prim Care.

[11] de Lusignan S, Mold F, Sheikh A, Majeed A, Wyatt JC, Quinn T, Cavill M, Gronlund TA, Franco C, Chauhan U, Blakey H, Kataria N, Barker F, Ellis B, Koczan P, Arvanitis TN, McCarthy M, Jones S, Rafi I (2014). Patients' online access to their electronic health records and linked online services: a systematic interpretative review. BMJ Open.

[12] Bärkås A, Scandurra I, Rexhepi H, Blease C, Cajander Å, Hägglund M (2021). Patients' access to their psychiatric notes: current policies and practices in Sweden. Int J Environ Res Public Health.

[13] Hägglund M, Scandurra I (2017). Patients' online access to electronic health records: current status and experiences from the implementation in Sweden. Stud Health Technol Inform.

[14] Grünloh C, Cajander Å, Myreteg G (2016). "The record is our work tool!"-physicians' framing of a patient portal in Sweden. J Med Internet Res.

[15] Hanna L, Gill SD, Newstead L, Hawkins M, Osborne RH (2017). Patient perspectives on a personally controlled electronic health record used in regional Australia. Health Inf Manag.

[16] Elers P, Nelson F (2018). Improving healthcare through digital connection? Findings from a qualitative study about patient portals in New Zealand. Aust J Prim Health.

[17] Riippa I, Linna M, Rönkkö I (2014). The effect of a patient portal with electronic messaging on patient activation among chronically ill patients: controlled before-and-after study. J Med Internet Res.

[18] Emont S (2011). Measuring the impact of patient portals: what the literature tells us. California Healthcare Foundation.

[19] Zanaboni P, Kummervold PE, Sørensen T, Johansen MA (2020). Patient use and experience with online access to electronic health records in Norway: results from an online survey. J Med Internet Res.

[20] Vodicka E, Mejilla R, Leveille SG, Ralston JD, Darer JD, Delbanco T, Walker J, Elmore JG (2013). Online access to doctors' notes: patient concerns about privacy. J Med Internet Res.

[21] Nazi KM, Turvey CL, Klein DM, Hogan TP, Woods SS (2015). VA OpenNotes: exploring the experiences of early patient adopters with access to clinical notes. J Am Med Inform Assoc.

[22] Moll J, Rexhepi H, Cajander Å, Grünloh C, Huvila I, Hägglund M, Myreteg G, Scandurra I, Åhlfeldt R-M (2018). Patients' experiences of accessing their electronic health records: national patient survey in Sweden. J Med Internet Res.

[23] Kruse CS, Argueta DA, Lopez L, Nair A (2015). Patient and provider attitudes toward the use of patient portals for the management of chronic disease: a systematic review. J Med Internet Res.

[24] Mold F, de Lusignan S (2015). Patients' online access to their primary care electronic health records and linked online services: implications for research and practice. J Pers Med.

[25] Louch G, Albutt A, Smyth K, O'Hara JK (2022). What do primary care staff think about patients accessing electronic health records? A focus group study. BMC Health Serv Res.

[26] (2017). Elektronische gegevensuitwisseling in de zorg. Ministerie van Volksgezondheid.

[27] OPEN = Signpost. OPEN.

[28] Rogers E (2003). Diffusions of Innovations.

[29] Batenburg R Healthcare professionals registries. Nivel.

[30] Your research: is it subject to the WMO or not?. CCMO Central Committee on Research Involving Human Subjects.

[31] Avdagovska M, Ballermann M, Olson K, Graham T, Menon D, Stafinski T (2020). Patient portal implementation and uptake: qualitative comparative case study. J Med Internet Res.

[32] DesRoches CM, Leveille S, Bell SK, Dong ZJ, Elmore JG, Fernandez L, Harcourt K, Fitzgerald P, Payne TH, Stametz R, Delbanco T, Walker J (2020). The views and experiences of clinicians sharing medical record notes with patients. JAMA Netw Open.

[33] Blease C, Torous J, Hägglund M (2020). Does patient access to clinical notes change documentation?. Front Public Health.

[34] Scandurra I, Jansson A, Forsberg-Fransson M-L, Ålander T (2017). Patient accessible EHR is controversial: lack of knowledge and diverse perceptions among professions. Int J Reliable Quality E Healthcare.

[35] Scandurra I, Jansson A, Forsberg-Fransson M, Ålander T (2015). Is ‘patient's online access to health records’ a good reform? – opinions from Swedish healthcare professionals differ. Procedia Comput Sci.

[36] Keplinger LE, Koopman RJ, Mehr DR, Kruse RL, Wakefield DS, Wakefield BJ, Canfield SM (2013). Patient portal implementation: resident and attending physician attitudes. Fam Med.

[37] Palen TE, Ross C, Powers JD, Xu S (2012). Association of online patient access to clinicians and medical records with use of clinical services. JAMA.

[38] Cajander Å, Moll J, Englund S, Hansman A (2018). Medical records online for patients and effects on the work environment of nurses. Stud Health Technol Inform.

[39] Miller DP, Latulipe C, Melius KA, Quandt SA, Arcury TA (2016). Primary care providers' views of patient portals: interview study of perceived benefits and consequences. J Med Internet Res.

[40] Turner A, Morris R, McDonagh L, Hamilton F, Blake S, Farr M, Stevenson F, Banks J, Atherton H, Rakhra D, Lasseter G, Feder G, Ziebland S, Hyde E, Powell J, Horwood J (2023). Unintended consequences of patient online access to health records: a qualitative study in UK primary care. Br J Gen Pract.

[41] Delbanco T, Walker J, Bell SK, Darer JD, Elmore JG, Farag N, Feldman HJ, Mejilla R, Ngo L, Ralston JD, Ross SE, Trivedi N, Vodicka E, Leveille SG (2012). Inviting patients to read their doctors' notes: a quasi-experimental study and a look ahead. Ann Intern Med.

[42] Fitton C, Fitton R, Hannan A, Fisher B, Morgan L, Halsall D (2014). The impact of patient record access on appointments and telephone calls in two English general practices: a population-based study. London J Prim Care (Abingdon).

[43] Thielmann RR, Hoving C, Schutgens-Kok E, Cals JW, Crutzen R (2023). Patient online access to general practice medical records: a qualitative study on patients' needs and expectations. Health Inf Manag.

