# Supplementary material for: General Practices’ Experiences With Patients’ Web-Based Access to Medical Records: Survey Study
Source: J Med Internet Res. 2023 Apr 7;25:e41832. doi: 10.2196/41832 (PMC10131748; doi:10.2196/41832)
Supplement: Multimedia Appendix 1 [file jmir_v25i1e41832_app1.pdf]

Multimedia Appendix

Online web survey questions about patient web-based access

Question 1a.

From 1 July 2020, it is legally required to provide patients electronic access to their own medical data. This concerns access to a summary of personal medical data, such as lab results, diagnosis of their general practitioner, allergies and medication.

Do you currently provide patients this web-based access to their medical record? Note: This does not concern e-consultations, video consultations, making appointments online and requesting repeat prescriptions online.

- Yes, I provide web-based access to my patients via participation in a regional coalition of the OPEN program.
- Yes, I provide web-based access to my patients on my own initiative, other than via participation in a regional coalition of the OPEN program.
- No, not yet, but I am planning to offer my patients web-based access.

Question 1b.

If you indicated ‘Yes’ in the question before, as of which year do you offer web-based access to your patients?

- 2010
- 2011
- 2012
- 2013
- 2014
- 2015
- 2016
- 2017
- 2018
- 2019
- 2020
- 2021

Question 2.
What are your own (first) experiences with providing your patients web-based access to their medical record?

- ○ Mostly positive
- ○ Mostly negative
- ○ Neutral, neither positive nor negative
- ○ I do not know (yet)

Question 3.
What are the (first) experiences of the other employees in your practice with the provision of web-based access? If you don't know the answer for your colleagues, you may want to ask them how they experience this, or you can choose the 'I do not know (yet)' option. If the displayed function does not (or has not had) to do with web-based access, then you do not fill in any answer for that function.

	Mostly positive	Mostly negative	Neutral, neither positive nor negative	I do not know (yet)
General practitioner(s)	○	○	○	○
General practice assistant(s)	○	○	○	○
Practice nurse(s) in somatic healthcare	○	○	○	○
Practice nurse(s) in mental healthcare	○	○	○	○
Practice manager(s)	○	○	○	○

Question 4. Could you please explain your answers to questions 2 and 3?

Question 5.
When a patient in your practice wants to use web-based access for the first time, how much time does your practice approximately spend on instruction and explanation per patient? For these questions, consider the situation in the past month. Note: This may be a difficult question to answer, so an estimation is sufficient. If you don't know how to answer this question, you can fill in 'I do not know (yet), I cannot (yet) estimate this'.

Minutes per patient:

____

- ○ I do not know (yet), I cannot (yet) estimate this.

Question 6.
What changes in the workload of your practice has web-based access led to since its provision? Note: This may be a difficult question to answer, so an estimation for each question is sufficient. If you don't know how to answer a question, you can fill in 'I do not know (yet)'. If a particular statement does not apply to your practice, you do not have to fill in an answer for that particular statement.

	Increased	Remained the same	Decreased	I do not know (yet)
The number of administrative actions	○	○	○	○
The number of patient questions about medical record changes	○	○	○	○
The number of consultations in your practice	○	○	○	○
The number of telephone consultations	○	○	○	○
The number of e-consultations	○	○	○	○
The number of video consultations	○	○	○	○
The number of referrals to secondary care	○	○	○	○
The number of referrals to mental healthcare	○	○	○	○

The number of referrals to the social domain	○	○	○	○
Time burden for the general practice owner(s)	○	○	○	○
Time burden for the other general practitioner(s)	○	○	○	○
Time burden for the general practice assistant(s)	○	○	○	○
Time burden for the practice manager(s)	○	○	○	○
Efficiency of the consultations	○	○	○	○
Quality of the consultations	○	○	○	○
Patients’ preparation for their consultation(s)	○	○	○	○
Patients’ understanding of their medical record	○	○	○	○
Use of 'layman’s language' by you and your colleagues	○	○	○	○
Staff alertness to the actuality of the medical record	○	○	○	○
Shared decision making with patients	○	○	○	○
The pleasure you and your colleagues experience in conducting consultations	○	○	○	○
Completeness of medication overview	○	○	○	○
Other changes, namely:	○	○	○	○

Question 7.
About what do patients contact your practice as a result of web-based access? (multiple answers possible):

- ☐ Episodes
- ☐ Diagnostic test results (e.g. lab results, imaging, blood test results)
- ☐ Notes from the consultation at your practice
- ☐ Notes from the telephone consultation with your practice
- ☐ Personal details (which are for example incorrect according to the patient)
- ☐ Allergy alerts
- ☐ Medication overview
- ☐ Correspondence (for example between the general practitioner and another healthcare professional)
- ☐ Evaluation and Plan in the patient's medical record
- ☐ Questions about how web-based access works
- ☐ Other, namely
- ☐ I do not know (yet), I cannot say (yet)

Question 8. Can you explain your answer to the previous question?
