# Supplementary material for: General Practices’ Experiences With Patients’ Web-Based Access to Medical Records: Survey Study
Source: J Med Internet Res. 2023 Apr 7;25:e41832. doi: 10.2196/41832 (PMC10131748; doi:10.2196/41832)
Supplement: Multimedia Appendix 2 [file jmir_v25i1e41832_app2.pdf]

Multimedia Appendix

Supplementary Tables – In-depth analyses regarding staff experiences with web-based access and effects of web-based access on general practice

Table S1. Staff experiences with the provision of web-based access (categorized by year of adoption), without the answer option *I do not know (yet)*.

Staff experiences	Overall sample[a]	Group 1 (<2020)	Group 2 (2020)	Group 3 (2021)	Test results
	% (n)	% (n)	% (n)	% (n)	X^2; *P*; *N*
**Respondent[b]**					χ^2_6=6.40; *P*=.17; *N*=392
Mostly positive	42% (178)	58% (23)	43% (97)	36% (46)	
Mostly negative	9% (39)	8% (3)	9% (19)	11% (14)	
Neutral	48% (204)	35% (14)	48% (107)	53% (69)	
Total	100% (421)	100% (40)	100% (233)	100% (129)	
**General practitioner**					χ^2_6=5.51; *P*=.24; *N*=360
Mostly positive	45% (174)	61% (23)	45% (92)	39% (46)	
Mostly negative	9% (33)	8% (3)	8% (16)	9% (10)	
Neutral	47% (180)	32% (12)	47% (97)	52% (61)	
Total	100% (387)	100% (38)	100% (205)	100% (117)	
**General practice assistant**					χ^2_6=10.04; *P*=.04; *N*=327
Mostly positive	42% (148)	59% (22)	43% (80)	36% (37)	
Mostly negative	13% (46)	19% (7)	12% (23)	13% (14)	
Neutral	44% (155)	22% (8)	45% (83)	51% (53)	
Total	100% (349)	100% (37)	100% (186)	100% (104)	
**Practice nurse in somatic health care**					χ^2_6=7.64; *P*=.11; *N*=280
Mostly positive	47% (142)	65% (20)	45% (72)	45% (40)	
Mostly negative	5% (15)	10% (3)	5% (8)	3% (3)	
Neutral	48% (143)	26% (8)	50% (80)	52% (46)	
Total	100% (300)	100% (31)	100% (160)	100% (89)	
**Practice nurse in mental health care**					χ^2_6=9.82; *P*=.04; *N*=186
Mostly positive	37% (73)	64% (14)	37% (38)	28% (17)	
Mostly negative	7% (13)	9% (2)	6% (6)	8% (5)	
Neutral	57% (112)	27% (6)	58% (60)	63% (38)	
Total	100% (198)	100% (22)	100% (104)	100% (60)	
**Practice manager**					χ^2_6=8.59; *P*=.07; *N*=211
Mostly positive	45% (101)	63% (15)	45% (53)	39% (27)	
Mostly negative	8% (18)	17% (4)	7% (8)	9% (6)	
Neutral	47% (107)	21% (5)	48% (56)	53% (37)	
Total	100% (225)	100% (24)	100% (117)	100% (70)	

[a] The percentages and numbers in the Overall sample column are not equal to the sum of the other 3 columns (based on the year of adoption), because not all respondents indicated when they started providing web-based access.

[b] From previous studies, we know that the respondent is generally a practice manager or practice owner.

Table S2. Effects of web-based access on the number of consultations, administrative actions and patient questions in general practices (categorized by year of adoption).

Type of contact/action	Overall sample[a]	Group 1 (<2020)	Group 2 (2020)	Group 3 (2021)	Test results
	% (n)	% (n)	% (n)	% (n)	*X*²; *P*; *N*
**e-Consultations**					χ^2_6=46.12; *P*<.001; *N*=438
Increased	66% (311)	90% (36)	74% (170)	51% (85)	
Remained the same	16% (76)	5% (2)	17% (38)	18% (30)	
Decreased	1% (3)	0% (0)	0% (0)	1% (1)	
I do not know (yet)	18% (83)	5% (2)	10% (22)	31% (52)	
Total	100% (473)	100% (40)	100% (230)	100% (168)	
**Administrative actions**					χ^2_6=18.89; *P*=.004; *N*=438
Increased	64% (302)	78% (31)	66% (152)	57% (97)	
Remained the same	21% (98)	15% (6)	21% (48)	22% (37)	
Decreased	2% (9)	0% (0)	3% (8)	1% (1)	
I do not know (yet)	14% (65)	8% (3)	9% (21)	21% (35)	
Total	100% (474)	100% (40)	100% (229)	100% (170)	
**Patient questions about medical record changes**					χ^2_6=36.72; *P*<.001; *N*=441
Increased	45% (215)	53% (21)	51% (119)	36% (60)	
Remained the same	33% (158)	43% (17)	36% (84)	29% (49)	
Decreased	0% (2)	0% (0)	0% (1)	1% (1)	
I do not know (yet)	21% (100)	5% (2)	12% (29)	35% (58)	
Total	100% (475)	100% (40)	100% (233)	100% (168)	
**Telephone consultations**					χ^2_6=27.30; *P*<.001; *N*=440
Increased	25% (120)	33% (13)	26% (59)	23% (39)	
Remained the same	46% (217)	46% (18)	53% (122)	38% (64)	
Decreased	10% (46)	13% (5)	10% (22)	9% (15)	
I do not know (yet)	19% (92)	8% (3)	12% (28)	31% (52)	
Total	100% (475)	100% (39)	100% (231)	100% (170)	
**Consultations in your practice**					χ^2_6=29.53; *P*<.001; *N*=442
Increased	12% (58)	18% (7)	15% (34)	7% (11)	
Remained the same	61% (291)	65% (26)	65% (151)	56% (95)	
Decreased	5% (22)	8% (3)	6% (13)	3% (5)	
I do not know (yet)	22% (106)	10% (4)	15% (35)	34% (58)	
Total	100% (477)	100% (40)	100% (233)	100% (169)	
**Video consultations**					χ^2_6=31.41; *P*<.001; *N*=419
Increased	10% (44)	13% (5)	12% (27)	5% (40)	
Remained the same	47% (211)	56% (22)	54% (119)	35% (197)	
Decreased	2% (7)	3% (1)	1% (3)	1% (2)	
I do not know (yet)	42% (188)	28% (11)	32% (71)	59% (94)	
Total	100% (450)	100% (39)	100% (220)	100% (160)	

[a] The percentages and numbers in the Overall sample column are not equal to the sum of the other 3 columns (based on the year of adoption), because not all participants indicated when they started providing web-based access.

Table S3. Effects of web-based access on specified general practice workflow processes (categorized by year of adoption).

Workflow process	Overall sample[a]	Group 1 (<2020)	Group 2 (2020)	Group 3 (2021)	Test results
	% (n)	% (n)	% (n)	% (n)	*X^2; P; N*
**Staff' alertness to the medical record's actuality**					χ^2_6=15.16; *P*=.02; *N*=439
Increased	50% (236)	55% (22)	52% (119)	46% (78)	
Remained the same	32% (153)	35% (14)	36% (82)	29% (49)	
Decreased	1% (4)	3% (1)	0% (1)	1% (1)	
I do not know (yet)	17% (80)	8% (3)	12% (28)	24% (41)	
Total	100% (473)	100% (40)	100% (230)	100% (169)	
**Use of *layman's language***					χ^2_6=22.67; *P*=.001; *N*=435
Increased	40% (188)	45% (18)	44% (101)	35% (58)	
Remained the same	40% (188)	43% (17)	44% (100)	35% (59)	
Decreased	2% (8)	0% (0)	1% (3)	2% (3)	
I do not know (yet)	18% (85)	13% (5)	11% (24)	28% (47)	
Total	100% (469)	100% (40)	100% (228)	100% (167)	
**Completeness of medication overview**					χ^2_6=24.07; *P*=.001; *N*=437
Increased	22% (105)	23% (9)	25% (57)	17% (29)	
Remained the same	56% (263)	65% (26)	60% (138)	50% (83)	
Decreased	2% (10)	5% (2)	2% (5)	2% (3)	
I do not know (yet)	20% (92)	8% (3)	13% (31)	31% (51)	
Total	100% (470)	100% (40)	100% (231)	100% (166)	
**Patients' preparation for their consultation**					χ^2_6=40.04; *P*<.001; *N*=437
Increased	18% (84)	23% (9)	22% (51)	10% (17)	
Remained the same	51% (238)	58% (23)	55% (126)	45% (75)	
Decreased	2% (9)	8% (3)	1% (3)	1% (2)	
I do not know (yet)	30% (140)	13% (5)	21% (49)	44% (74)	
Total	100% (471)	100% (40)	100% (229)	100% (168)	
**Patients' understanding of their medical record**					χ^2_6=32.99; *P*<.001; *N*=440
Increased	16% (76)	20% (8)	20% (47)	9% (15)	
Remained the same	43% (206)	58% (23)	48% (111)	35% (59)	
Decreased	6% (27)	8% (3)	4% (10)	7% (12)	
I do not know (yet)	35% (165)	15% (6)	28% (64)	49% (82)	
Total	100% (474)	100% (40)	100% (232)	100% (168)	
**Shared decision making with patients**					χ^2_6=23.92; *P*=.001; *N*=435
Increased	14% (67)	25% (10)	14% (32)	11% (18)	
Remained the same	63% (293)	63% (25)	69% (158)	56% (93)	
Decreased	1% (5)	0% (0)	1% (3)	1% (1)	
I do not know (yet)	22% (103)	13% (5)	15% (35)	33% (55)	
Total	100% (468)	100% (40)	100% (228)	100% (167)	
**Efficiency of consultations**					χ^2_6=45.06; *P*<.001; *N*=439

Increased	12% (59)	10% (4)	16% (38)	7% (12)	
Remained the same	55% (259)	70% (28)	57% (132)	49% (81)	
Decreased	8% (37)	15% (6)	10% (6)	4% (6)	
I do not know (yet)	25% (118)	5% (2)	17% (40)	40% (67)	
Total	100% (473)	100% (40)	100% (233)	100% (166)	
**Quality of consultations**					χ^2_6=38.33; *P*<.001; *N*=436
Increased	10% (49)	10% (4)	13% (30)	5% (9)	
Remained the same	60% (283)	77% (30)	64% (148)	52% (86)	
Decreased	4% (21)	8% (3)	6% (13)	3% (5)	
I do not know (yet)	25% (117)	5% (2)	17% (40)	40% (66)	
Total	100% (470)	100% (39)	100% (231)	100% (166)	
**Other changes, namely**					χ^2_6=3.71; *P*=.72; *N*=114
Increased	17% (23)	38% (3)	12% (6)	14% (8)	
Remained the same	16% (21)	13% (1)	16% (8)	14% (8)	
Decreased	2% (2)	0% (0)	2% (1)	2% (1)	
I do not know (yet)	65% (86)	50% (4)	70% (35)	70% (39)	
Total	100% (132)	100% (8)	100% (50)	100% (56)	
**Pleasure in conducting consultations**					χ^2_6=22.14; *P*=.001; *N*=433
Increased	4% (21)	5% (2)	5% (11)	2% (4)	
Remained the same	59% (277)	63% (25)	63% (143)	55% (92)	
Decreased	16% (74)	20% (8)	18% (40)	11% (18)	
I do not know (yet)	21% (96)	13% (5)	14% (32)	32% (53)	
Total	100% (468)	100% (40)	100% (226)	100% (167)	

[a] The percentages and numbers in the Overall sample column are not equal to the sum of the other 3 columns (based on the year of adoption), because not all participants indicated when they started providing web-based access.

Table S4. Effects of web-based access on time burden in general practices, specified by function (categorized by year of adoption).

General practice staff member	Overall sample[a]	Group 1 (<2020)	Group 2 (2020)	Group 3 (2021)	Test results
	% (n)	% (n)	% (n)	% (n)	*X^2; P; N*
**General practice owner(s)**					χ^2_6=29.84; *P*<.001; *N*=443
Increased	59% (284)	79% (31)	65% (151)	48% (82)	
Remained the same	22% (106)	15% (6)	23% (54)	23% (40)	
Decreased	1% (7)	3% (1)	1% (3)	1% (2)	
I do not know (yet)	17% (81)	5% (2)	10% (24)	27% (47)	
Total	100% (478)	100% (40)	100% (232)	100% (171)	
**General practice assistant(s)**					χ^2_6=33.68; *P*<.001; *N*=442
Increased	51% (243)	65% (26)	53% (122)	47% (80)	
Remained the same	25% (120)	18% (7)	27% (63)	24% (40)	
Decreased	8% (38)	13% (5)	11% (25)	3% (5)	
I do not know (yet)	16% (76)	8% (3)	9% (22)	26% (45)	
Total	100% (477)	100% (40)	100% (232)	100% (170)	
**Other general practitioner(s)**					χ^2_6=20.89; *P*=.002; *N*=425
Increased	37% (172)	65% (20)	40% (88)	30% (50)	
Remained the same	37% (172)	18% (14)	41% (91)	35% (58)	
Decreased	2% (7)	13% (1)	2% (24)	1% (1)	
I do not know (yet)	24% (108)	8% (5)	17% (37)	34% (56)	
Total	100% (459)	100% (40)	100% (220)	100% (165)	
**Practice manager(s)**					χ^2_6=14.18; *P*=.03; *N*=425
Increased	34% (156)	44% (17)	35% (76)	30% (50)	
Remained the same	31% (142)	26% (10)	35% (78)	27% (44)	
Decreased	1% (5)	3% (1)	1% (3)	0% (0)	
I do not know (yet)	34% (155)	28% (11)	29% (63)	43% (72)	
Total	100% (458)	100% (39)	100% (220)	100% (166)	

[a] The percentages and numbers in the Overall sample column are not equal to the sum of the other 3 columns (based on the year of adoption), because not all participants indicated when they started providing web-based access.

Table S5. What are practices contacted about because of the provision of web-based access? (categorized by year of adoption).

Item	Overall sample[a]	Group 1 (<2020)	Group 2 (2020)	Group 3 (2021)	Test results
	% (n)	% (n)	% (n)	% (n)	X^2; *P*; *N*
**Diagnostic test results**					χ^2_2=19.57; *P*<.001; *N*=445
Yes	70% (336)	83% (33)	76% (179)	58% (99)	
No	30% (146)	18% (7)	24% (55)	42% (72)	
Total	100% (482)	100% (40)	100% (234)	100% (171)	
**Episode lists**					χ^2_2=12.62; *P*=.002; *N*=445
Yes	48% (230)	65% (26)	53% (111)	39% (66)	
No	52% (252)	35% (14)	47% (123)	61% (105)	
Total	100% (482)	100% (40)	100% (234)	100% (171)	
**Medication overview**					χ^2_2=15.21; *P*<.001; *N*=445
Yes	43% (206)	58% (23)	48% (113)	32% (54)	
No	57% (276)	43% (17)	52% (121)	68% (117)	
Total	100% (482)	100% (40)	100% (234)	100% (171)	
**Questions about how online access works**					χ^2_2=5.01; *P*=.08; *N*=445
Yes	42% (201)	55% (22)	45% (106)	37% (64)	
No	58% (281)	45% (18)	55% (128)	63% (107)	
Total	100% (482)	100% (40)	100% (234)	100% (171)	
**Personal details**					χ^2_2=16.45; *P*<.001; *N*=445
Yes	30% (145)	58% (23)	31% (73)	25% (42)	
No	70% (337)	43% (17)	69% (161)	75% (129)	
Total	100% (482)	100% (40)	100% (233)	100% (169)	
**Evaluation and Plan in the patient's medical record**					χ^2_2=3.39; *P*=.18; *N*=445
Yes	18% (88)	23% (9)	20% (46)	13% (23)	
No	82% (394)	78% (31)	80% (188)	87% (148)	
Total	100% (482)	100% (40)	100% (234)	100% (171)	
**Allergy alerts**					χ^2_2=6.72; *P*=.04; *N*=445
Yes	18% (88)	25% (10)	22% (52)	13% (22)	
No	82% (394)	75% (30)	78% (182)	87% (149)	
Total	100% (482)	100% (40)	100% (234)	100% (171)	
**Correspondence**					χ^2_2=14.61; *P*=.001; *N*=445
Yes	17% (83)	35% (14)	18% (41)	11% (18)	
No	83% (399)	65% (26)	82% (193)	89% (153)	
Total	100% (482)	40 (100%)	234 (100%)	171 (100%)	
**I do not know (yet), I cannot say (yet)**					χ^2_2=33.76; *P*<.001; *N*=445
Yes	17% (82)	5% (2)	9% (21)	29% (50)	

No	83% (400)	95% (38)	91% (213)	71% (121)	
Total	100% (482)	100% (40)	100% (234)	100% (171)	
**Notes from the consultation at your practice**					χ^2_2=6.26; *P*=.04; *N*=445
Yes	13% (61)	18% (7)	16% (38)	8% (14)	
No	87% (421)	83% (33)	84% (196)	92% (157)	
Total	100% (482)	100% (40)	100% (234)	100% (171)	
**Notes from a telephone consultation**					χ^2_2=6.59; *P*=.04; *N*=445
Yes	8% (40)	15% (6)	11% (25)	5% (8)	
No	92% (442)	85% (34)	89% (209)	95% (163)	
Total	100% (482)	100% (40)	100% (234)	100% (171)	
**Other, namely**					χ^2_2=1.35; *P*=.51; *N*=445
Yes	7% (33)	3% (1)	7% (16)	8% (13)	
No	93% (449)	98% (39)	93% (218)	92% (158)	
Total	100% (482)	100% (40)	100% (234)	100% (171)	

[a] The percentages and numbers in the Overall sample column are not equal to the sum of the other 3 columns (based on the year of adoption), because not all participants indicated when they started providing web-based access.
